# Genome Sequence of Geobacillus thermoleovorans SGAir0734, Isolated from Singapore Air

**DOI:** 10.1128/genomeA.00636-18

**Published:** 2018-07-05

**Authors:** Nicolas E. Gaultier, Ana Carolina M. Junqueira, Akira Uchida, Rikky W. Purbojati, James N. I. Houghton, Caroline Chénard, Anthony Wong, Sandra Kolundžija, Megan E. Clare, Kavita K. Kushwaha, Deepa Panicker, Alexander Putra, Carmon Kee, Balakrishnan N. V. Premkrishnan, Cassie E. Heinle, Serene B. Y. Lim, Vineeth Kodengil Vettath, Daniela I. Drautz-Moses, Stephan C. Schuster

**Affiliations:** a Singapore Centre for Environmental Life Sciences Engineering, Nanyang Technological University, Singapore; b Asian School of the Environment, Nanyang Technological University, Singapore

## Abstract

The thermophilic bacterium Geobacillus thermoleovorans was isolated from a tropical air sample collected in Singapore. The genome was sequenced on the PacBio RS II platform and consists of one chromosome with 3.6 Mb and one plasmid with 75 kb. The genome comprises 3,509 protein-coding genes, 88 tRNAs, and 27 rRNAs.

## Abstract (precis)

The thermophilic bacterium Geobacillus thermoleovorans was isolated from a tropical air sample collected in Singapore. The genome was sequenced on the PacBio RS II platform and consists of one chromosome with 3.6 Mb and one plasmid with 75 kb.

## GENOME ANNOUNCEMENT

Geobacillus thermoleovorans is a rod-shaped Gram-positive thermophilic bacterium (1) classified in the phylum *Firmicutes*. It was first reported as strain LEH-1 in a study on heat sensitivity of thermophilic bacteria (2) and later designated Bacillus thermoleovorans (3). Geobacillus thermoleovorans is found in a variety of environments, including gold mines (4), sugar refineries (5), and hot springs (6). This organism’s thermophilic characteristic makes it interesting for potential industrial applications (7).

SGAir0734 was isolated from an air sample collected in Singapore (global position system coordinates 1.345771°N, 103.6801°E) using the Spin Air air sampler (IUL, Spain). The airborne particles were impacted onto Reasoner’s 2A agar (Becton, Dickinson). Isolation of colonies was carried out by culturing on Trypticase soy agar (Becton, Dickinson) at 50°C and in Luria broth overnight at 30°C prior to DNA extraction. Genomic DNA was purified using the Wizard genomic DNA purification kit (Promega, USA) according to the manufacturer’s protocol. Library preparation was performed with the SMRTbell template prep kit 1.0 (Pacific Biosciences), followed by single-molecule real-time (SMRT) sequencing on the PacBio RS II platform.

Sequencing resulted in a total of 55,892 subreads. The reads were subsequently used for *de novo* assembly with Hierarchical Genome Assembly Process (HGAP) version 3 (8), which is included in the PacBio SMRT Analysis 2.3.0 package. In order to polish and correct the errors of the assembly, Quiver (8) and Pilon version 1.16 (9) were used. The consensus assembly generated two contigs, including one chromosome with 3,615,819 bp (146-fold coverage) and one plasmid, unnamed_3, with 75,441 bp (259-fold coverage). Both contigs were unable to be circularized using Circlator (10). The average chromosome G+C content was 52.19%.

Using the average nucleotide identity (ANI) method with Microbial Species Identifier (MiSI) (11), taxonomical identification revealed a 99.0% marker similarity with the available reference genome of Geobacillus thermoleovorans.

Annotation was completed using NCBI’s Prokaryotic Genome Annotation Pipeline (PGAP) version 4.2 (12). A total of 3,850 genes were predicted, including 3,509 protein-coding genes (PCGs), 27 rRNA subunits (9 genes for each of the 5S, 16S, and 23S subunits), 88 tRNAs, 5 noncoding RNAs, and 221 pseudogenes. Functional annotation with Rapid Annotations using Subsystems Technology (RAST) (13–15) showed that 116 genes were associated with dormancy and sporulation, which could indicate the ability to enter long-term survival states in hostile environments. Seventeen genes were found to be potentially involved in stress response, specifically heat shock, which may contribute to the thermophilicity of this organism.

### Accession number(s).

The genome and plasmid sequences of Geobacillus thermoleovorans SGAir0734 are available in DDBJ/EMBL/GenBank under accession numbers CP027303 and CP027305, respectively.
